# Supplementary material for: Establishment of a Cell Culture Model of Persistent Flaviviral Infection: Usutu Virus Shows Sustained Replication during Passages and Resistance to Extinction by Antiviral Nucleosides
Source: Viruses. 2019 Jun 17;11(6):560. doi: 10.3390/v11060560 (PMC6630443; doi:10.3390/v11060560)
Supplement: Supplementary file 1 [file viruses-11-00560-s001.pdf]

Supplementary material

Establishment of a cell culture model of persistent flaviviral infection: Usutu virus shows sustained replication during passages and resistance to extinction by antiviral nucleosides

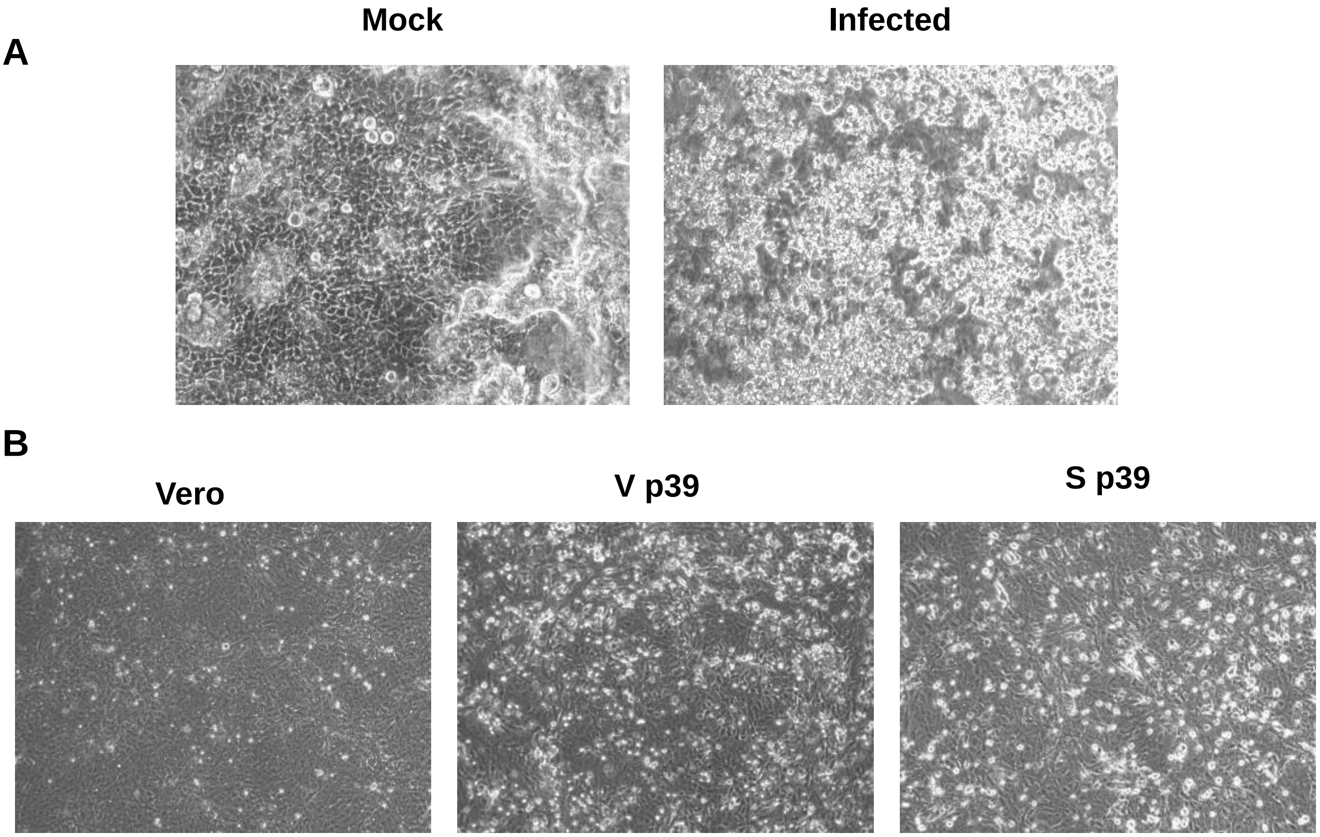

Figure S1. Cellular monolayers of Vero cells infected with USUV. A, Lytic infection (right) compared to Mock-infected cells (left) in 96-well plates. Mock cells show multilayer formations (right). B, Monolayers of uninfected Vero, V p39 and S p39 at day 5 after seeding in 75 cm2 flasks.

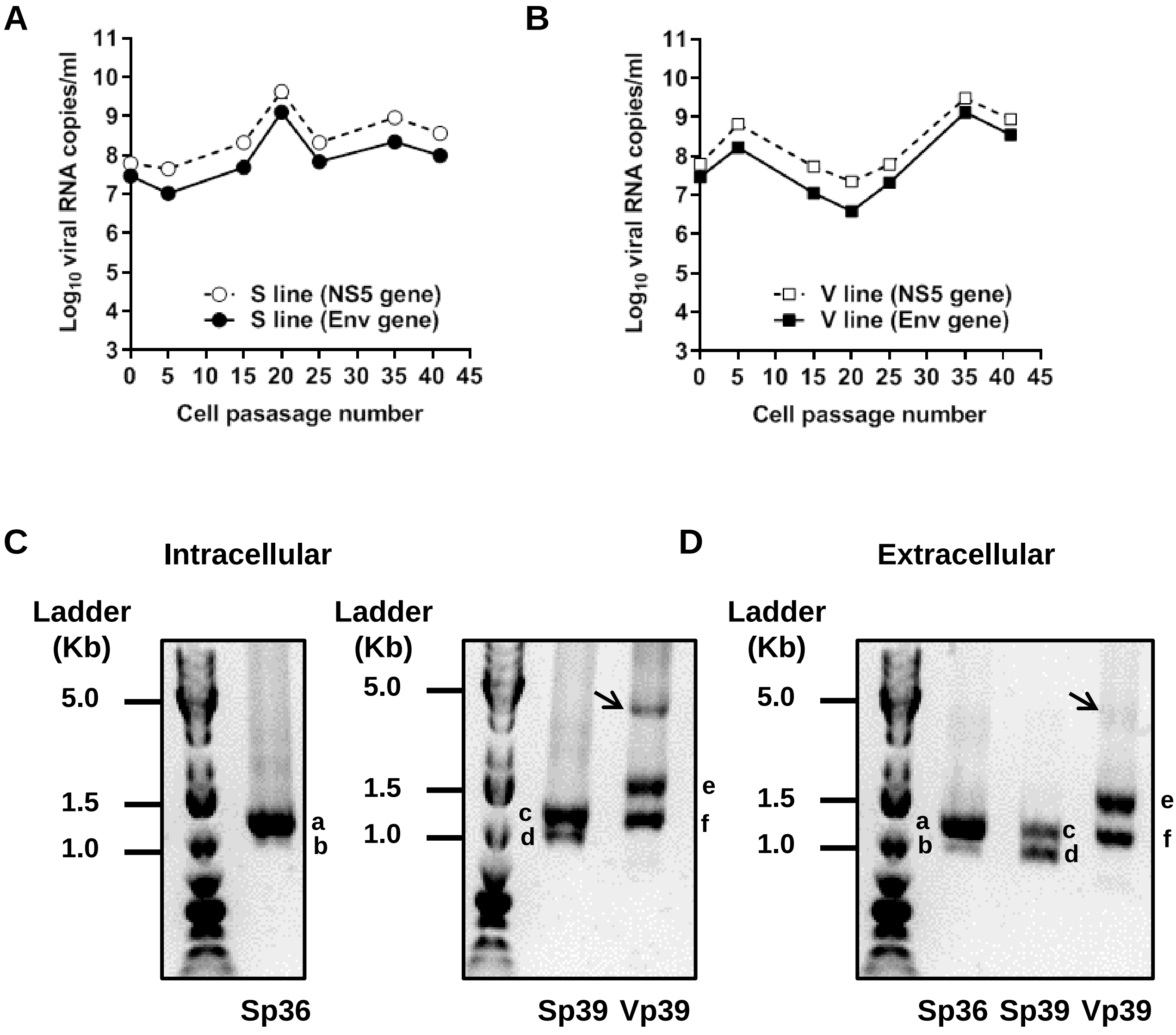

Figure S2. Viral RNA in cell supernatants along passages. The number of molecules was determined by qPCR, using primers for the specific detection of Env- (black symbols) or NS5-coding regions (white symbols). C-D, Amplicons identified in samples obtained from persistently-infected cells V (passage 39, V p39) and S (passages 36 and 39, S p36 and S p39) using primers spanning residues 1 to 3359. C, PCR amplification of viral RNA extracted from cells. D, PCR amplification of viral RNA extracted from cellular supernatants after micrococcal nuclease treatment to eliminate non-encapsidated genomes, as previously described (Arias et al 2014 eLife; ;3:e03679). Different amplicon lengths detected in V p39 and S p39 intracellular and extracellular RNA extracts are indicated with a lowercase letter (a to f). An arrow indicate the detection of full-length genome amplicons.

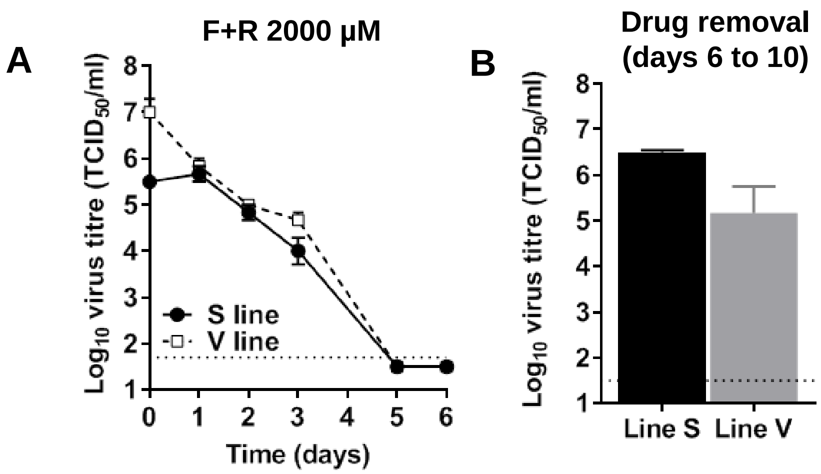

Figure S3. Prolonged exposure of persistently-infected cells to a cocktail of FAV and RBV eliminates viral infectivity in the supernatant. A, Continuous treatment of V and S cells at passage 34 with a combination of FAV and RBV (F+R) at a concentration of 2000 µM each, leads to undetectable levels of infectious virus. Cells were treated with F+R during 6 days. At day 3, cellular supernatants were removed and fresh media containing F+R was added to the cells. B, At day 6, drugs were removed and the cells cultured for 4 additional days. Removal of drug treatment leads to a relapse in virus titres in the supernatant of both V and S cells.
